# Targeting copper metabolism: a promising strategy for cancer treatment

**DOI:** 10.3389/fphar.2023.1203447

**Published:** 2023-07-26

**Authors:** Ruimin Kong, Guojuan Sun

**Affiliations:** ^1^ School of Medical and Life Sciences, Chengdu University of Traditional Chinese Medicine, Chengdu, Sichuan Province, China; ^2^ Hospital of Chengdu University of Traditional Chinese Medicine, Chengdu, Sichuan Province, China

**Keywords:** copper, cuprotosis, cancer treatment, metabolism, tumor microenvironment

## Abstract

Copper is an essential micronutrient that plays a critical role in many physiological processes. However, excessive copper accumulation in cancer cells has been linked to tumor growth and metastasis. This review article explores the potential of targeting copper metabolism as a promising strategy for cancer treatment. Excessive copper accumulation in cancer cells has been associated with tumor growth and metastasis. By disrupting copper homeostasis in cancer cells and inducing cell death through copper-dependent mechanisms (cuproplasia and cuprotosis, respectively), therapies can be developed with improved efficacy and reduced side effects. The article discusses the role of copper in biological processes, such as angiogenesis, immune response, and redox homeostasis. Various approaches for targeting copper metabolism in cancer treatment are examined, including the use of copper-dependent enzymes, copper-based compounds, and cuprotosis-related genes or proteins. The review also explores strategies like copper chelation therapy and nanotechnology for targeted delivery of copper-targeting agents. By understanding the intricate network of cuprotosis and its interactions with the tumor microenvironment and immune system, new targets for therapy can be identified, leading to improved cancer treatment outcomes. Overall, this comprehensive review highlights the significant potential of targeting copper metabolism as a promising and effective approach in cancer treatment, while providing valuable insights into the current state of research in this field.

## Introduction

Copper is an essential micronutrient that plays a critical role in many physiological processes, including cellular growth and proliferation. Copper is required for the activity of enzymes involved in DNA synthesis, cell division, and angiogenesis, making it a key player in the regulation of cellular proliferation. However, excessive copper accumulation in cancer cells has been linked to tumor growth and metastasis. Recent studies have shown that targeting copper metabolism has the potential to revolutionize cancer therapy by inducing cuproplasia and cuprotosis, which refer to the disruption of copper homeostasis in cancer cells and the induction of cell death through copper-dependent mechanisms, respectively.

Cell death is a fundamental process in the maintenance of homeostasis and the development of multicellular organisms. It can be divided into accidental cell death (ACD), which is caused by severe physical, chemical, or mechanical damage, and programmed cell death (PCD), which is a highly regulated process involved in various biological phenomena (Meier et al., 2000). However, malignant cells have endogenous resistance to apoptosis, allowing them to survive and proliferate uncontrollably. Non-apoptotic regulated cell death (NARCD) is an alternative form of cell death that different with apoptosis and its typical biochemical pathways, such as necrotosis, ferroptosis and cuprotosis (Yu et al., 2021; Li et al., 2020). These pathways can be regulated even if the cancer cells are resistant to apoptosis, making NARCD a potential target for cancer treatment (Okada and Mak, 2004).

In 2012, Ferroptosis was firstly proposed, an iron-dependent, non-apoptotic mode of cell death which showed obvious mitochondria shrinkage and increased membrane density (Friedmann Angeli et al., 2014). This is different with typical morphological characteristics of necrosis (cytoplasm swelling and cell membrane) and apoptosis (chromatin condensation and formation of apoptotic bodies). Circulating iron binds to transferrin in the form of Fe3+ and is transported into cells via the transferrin receptor 1. Iron can be stored in ferritin, a protein that binds iron in a safe and non-reactive form, thereby preventing it from participating in reactive oxygen species (ROS) generation reactions (Xie et al., 2016). Excess iron is the base of ferroptosis. The essence of ferroptosis is the exhausting of glutathione and glutathione peroxidase downregulation (Tang and Kroemer, 2020). In this condition, the lipid oxide can’t be metabolized, and Fe2+ is passed through Fenton reaction oxidizes lipids to generate ROS (Jiang et al., 2021). Recent research has demonstrated that ferroptosis is a crucial regulatory mechanism in the onset and progression of numerous diseases, making it a major area of interest in improving the treatment and prognosis of these conditions (Xu et al., 2022).

Unlike the ferropotosis which is characterized by iron-dependent accumulation of lipid peroxides, another new type of cell death named cuprotosis is found to be associated with accumulation of copper ions and the disruption of cellular metabolism. The main process of cuprotosis involves the accumulation of copper in cells, which directly bind to fatty acylation components in the tricarboxylic acid cycle (TCA cycle) (Tsvetkov et al., 2022). This leads to the aggregation and imbalance of these proteins, blocking the TCA cycle, inducing protein toxic stress, and ultimately resulting in cell death. Numerous studies showed that accumulation of copper ions can cause cytotoxicity, increasing the intracellular copper concentration can induce specific killing effect (Kahlson and Dixon, 2022). We will explore the intricate interplay between copper metabolism and cancer, with a particular emphasis on the potential applications of copper in cancer treatment.

## Role of copper in biological system

**FIGURE 1:**
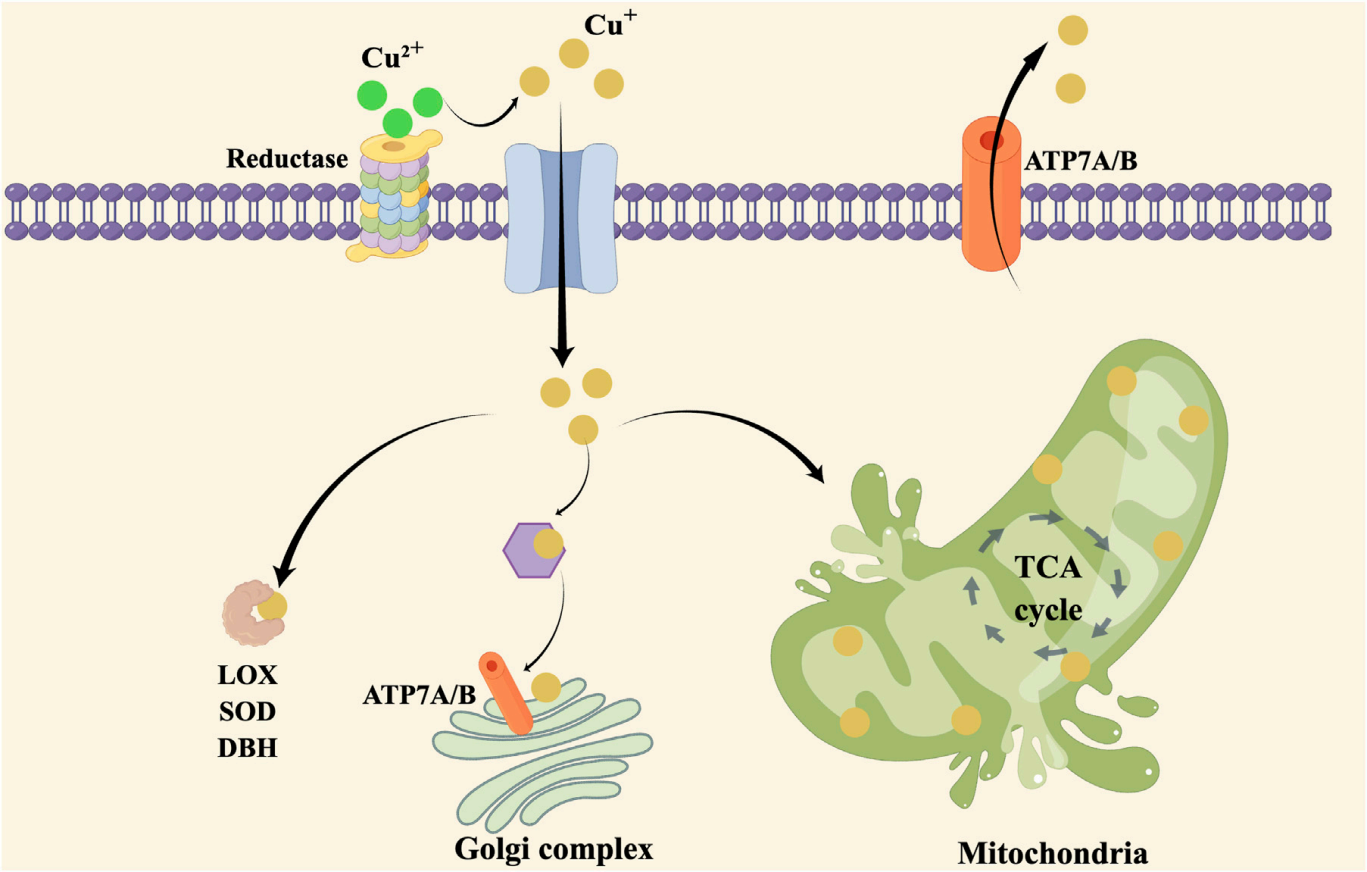
Copper hemostasis and cuprotosis in cells.

Copper plays a crucial role in enzymatic reactions, mitochondrial function and antioxidant defense. The regulation of copper homeostasis is critical to prevent both deficiency and excess levels of copper. Copper transporters and chaperones, such as ATP7A and ATP7B, play a crucial role in maintaining copper homeostasis by facilitating copper uptake, transport, and excretion in different tissues. Copper metabolism abnormalities refer to the conditions in which there is an imbalance in copper levels, leading to either copper deficiency or overload. Cuproplasia and cuprotosis are both increased level of copper found in cancer cells which is important for metabolism and targeting therapy. The copper metabolism and cuprotosis are shown in Figure 1.

### Copper homeostasis

It refers to the balance and regulation of copper levels in the body. Copper is an essential mineral that plays a critical role in various cellular functions, including enzyme regulation, mitochondrial respiration, and antioxidant defense (Burkhead et al., 2009). Copper misbalance affects central nervous system, liver function, lipid metabolism and resistance to chemotherapy (Gaetke et al., 2014). Copper in the diet is mainly stored in the muscular and bone tissues, with 20% stored in the liver and 10% in the blood (Miranda et al., 2010). Copper is absorbed in the form of Cu2+ and then reduced to Cu + by reductases on the surface of epithelial cells in the digestive tract. Cu+ is then transported into cells via copper transporter 1 (CTR1) and combined with copper chaperone protein antioxidant-1 (Atox1) to enter hepatocytes through copper transporting ATPase β (ATP7B) Golgi complex pathway to form ceruloplasmin (Kamiya et al., 2018). The distribution of ATR1 in cells is dynamic, elevation of extracellular copper induces endocytosis of CTR1 to vesicles, whereas a decrease in extracellular cooper restores CTR1 (Lutsenko, 2010). Excessive copper is excreted into bile in the form of vesicles by ATP7B, while ATP7A can mobilize copper from liver storage to maintain effective copper concentration (Polishchuk Elena et al., 2014). Copper deficiency can lead to oxidative stress and cytotoxicity, while copper excess can also be toxic. Menkes disease is a rare genetic disorder that results in copper deficiency due to mutations in the ATP7A gene (Kaler, 2011).

### Cuproplasia

It is a term used to describe the role of copper in promoting cell growth and proliferation. This process is regulated by signaling pathways and can involve both enzymatic and non-enzymatic activities of copper (Ge et al., 2022). Cuproplasia can lead to the development of neoplasia or hyperplasia, and can be targeted using copper-selective chelators or metal ionophores. Additionally, proteins involved in copper homeostasis can be genetically or pharmacologically manipulated to modulate cuproplasia. It refers to a condition in which there is an abnormal accumulation of copper in tissues or organs due to a genetic or metabolic disorder. It is a pathological condition that results from an imbalance in copper homeostasis. Cuproplasia is linked to several cellular processes, such as mitochondrial respiration, redox signaling, autophagy, antioxidant defence and kinase signaling (Ge et al., 2022).

### Cuprotosis

In 2022, Todd’s et al. found copper directly binds to thioctylated components of the TCA cycle and leads to the downregulation of Fe-S cluster proteins and abnormal aggregation of thiotylated proteins, resulting in a distinct form of cell death named “cuprotosis” which is a significant contribution to our understanding of cell death mechanisms (Tsvetkov et al., 2022). This process involves increased mitochondrial energy metabolism and the accumulation of reactive oxygen species, ultimately leading to cell death. The fact that knocking down Bax BAK1 did not obstruct copper-induced cell death suggests that cuprotosis is distinct from apoptosis, which involves the activation of Bax BAK1.

Further studies on the mechanism of cuprotosis, exploring the relationship between ferroptosis and cuprotosis. In colon cancer cells treated with a combination of elesclomol (an anticancer drug that targets mitochondrial metabolism) and copper, it was found that copper accumulation in the mitochondria led to an increase in reactive oxygen species and the solute carrier family 7 member 11 (SLC7A11) protein, which is involved in the regulation of cellular antioxidant response (Gao et al., 2021). However, subsequent studies found that mitochondrial antioxidants and inhibitors of mitochondrial function were more effective at inhibiting cell death induced by elesclomol than ferroptosis inhibitors (Tsvetkov et al., 2022). Moreover, studies have shown that prolonged use of elesclomol can lead to an increase in the TCA cycle metabolites produced by non-small cell lung cancer cells, suggesting that the target of copper-induced cell death may be the TCA cycle process itself (Zheng P. et al., 2022). Based on these latest researches, cuprotosis does not seem to be a copper-dependent form of ferroptosis. Instead, it appears to target the TCA cycle process rather than the electron transport chain process. This suggests that targeting mitochondrial metabolism, rather than the regulation of cellular antioxidant response, may be a more effective strategy for preventing cuprotosis in cancer cells.

Furthermore, Multiple CRISPR knockout screens were performed to identify cuprotisis-relating gens. The result showed FDX1, LIAS, LIPT1, DLD, DLAT, PDHA1, and PDHB are positively regulated genes. On the other hand, MTF1, GLS, and CDKN2A were confirmed as negatively genes (Tsvetkov et al., 2022). Of these genes, FDX1 and protein lipoylation were identified as key factors of cuprotosis genes led to enhanced resistance to cuprotosis, indicating a strong connection between FDX1, protein lipoylation, and cuprotosis (Wang et al., 2022; Schulz et al., 2023; Shen et al., 2023).

## Mechanism of targeting copper or cuprotosis in cancer treatment

**FIGURE 2:**
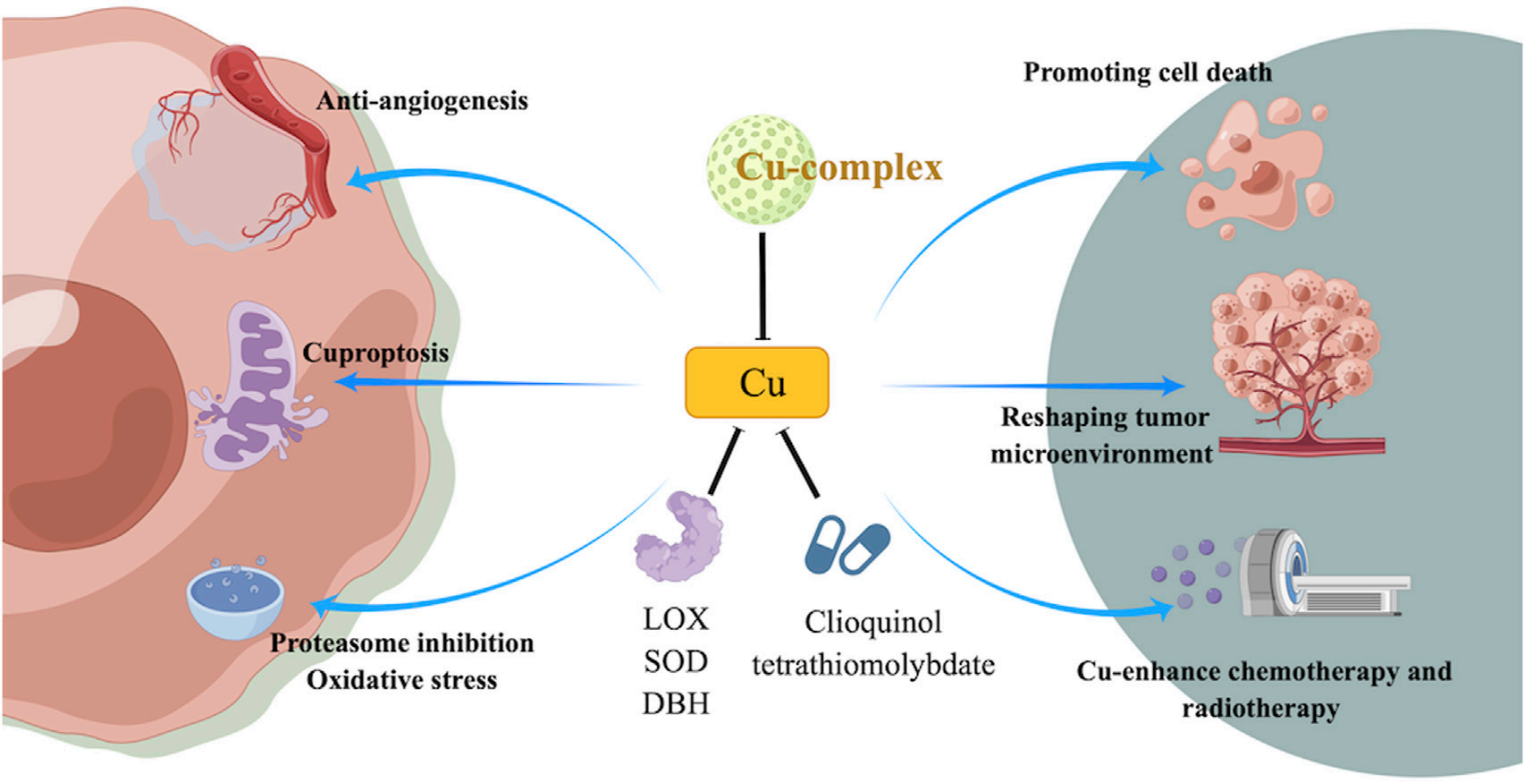
Mechanism of targeting copper or cuprotosis and its potential role in targeted therapy of cancer treatment.

Copper is an important element that plays an important role in many cellular processes, including angiogenesis and enzyme activity. However, in cancer cells, copper can be dysregulated and contribute to tumor growth and survival. Inhibiting copper-dependent angiogenesis, inducing cancer cell death by disrupting copper balance, and reducing the amount of copper available to cancer cells are potential strategies for inhibiting tumor growth and enhancing sensitivity to chemotherapy and radiotherapy. Additionally, copper may also play a role in reshaping the tumor microenvironment and enhancing immunotherapy (Figure 2).

### Inhibiting tumor growth and promoting cell death

Copper plays a crucial role in promoting tumor growth by promoting angiogenesis. It is involved in the activity of several angiogenic factors, including vascular endothelial growth factor (VEGF) and angiogenin. Copper ions can bind to VEGF and promote its dimerization, which is required for VEGF to bind to its receptor and initiate signaling pathways that promote angiogenesis. Therefore, blocking copper-dependent angiogenesis is a potential strategy for inhibiting tumor growth. The copper participates in angiogenesis in two aspects: 1) it interacts with cellular matrix with an increased redox potential to product oxidative products, then revealing DNA mutations in the nucleus and mitochondria or alternations to membrane phospholipids; 2) it regulates angiogenesis even in the absence of angiogenic molecules (Antoniades et al., 2013). Study found that the copper chelator inhibited the growth of pancreatic cancer cells *in vitro* and *in vivo* by inducing apoptosis and reducing the levels of copper-dependent angiogenic factors such as VEGF (Zhao et al., 2020).

In addition to their anti-angiogenic effects, copper chelators can also induce cancer cell death by disrupting the balance of copper ions in cells (Lowndes and Harris, 2005). Copper is required for the activity of several enzymes that regulate cellular processes, and an excess of copper can lead to oxidative stress and damage to cellular components (Antoniades et al., 2013). Furthermore, it can reduce the levels of copper ions in cells, leading to the activation of apoptotic pathways and ultimately inducing cancer cell death (Finney et al., 2009).

### Enhancing sensitivity to chemotherapy and radiotherapy

Cancer cells have been shown to have higher copper levels compared to normal cells. Thus, by reducing the amount of copper available to cancer cells, it is possible to sensitize them to chemotherapy and radiotherapy. A study reported that copper depletion sensitized ovarian cancer cells to radiation therapy by increasing the production of reactive oxygen species and inducing DNA damage (Kim et al., 2012). Similarly, another study found that copper chelation enhanced the effectiveness of the chemotherapy drug cisplatin in lung cancer cells by inducing oxidative stress and inhibiting DNA repair mechanisms (Nechushtan et al., 2015). Combination of anti-copper drugs and chemotherapy or radiotherapy are now becoming a new strategy and there are several preclinical studies showed promising results in melanoma, colorectal cancer and (Gartner et al., 2009; Ikeda et al., 2011; Grimaldi et al., 2017).

### Enhancing immunotherapy and reshaping tumor microenvironment

Some studies have suggested that copper may play a role in enhancing the efficacy of immunotherapy. One potential mechanism by which copper may enhance immunotherapy is through the release of damage-associated molecular patterns (DAMPs) from dying cancer cells. DAMPs are molecules that are released by dying cells and can stimulate the immune system to mount an anti-tumor response. In addition, copper treatment may also enhance the activity of immune cells, such as T cells and natural killer cells, which can recognize and eliminate cancer cells (Hu et al., 2020). Study showed that a copper complex (Cu-Cy NPs) could enhance checkpoint blockade melanoma immunotherapy (Zhang et al., 2020).

There are limited studies on cuprotosis and immunotherapy. Integrative analysis showed cuprotosis reshaped tumor microenvironment and response to immunotherapy of colorectal cancer by collecting 1,226 samples for genomic testing (Xu et al., 2023). In another study, Cuprotosis-related LncRNA models was constructed in liver cancer samples. The results showed six differentially expressed immune functions were found it high and low-risk groups. And the survival rate is significantly worse in high mutation groups compared with low risk groups (Chen et al., 2023). With the same technology in pancreatic ductal adenocarcinoma patients, Some LncRNA s are thought to have strong correlations with ductal adenocarcinoma outcomes, including AC005332.6, LINC02041, LINC00857, and AL117382 (Chi et al., 2022). The same results in lung adenocarcinoma were seen (Zheng M. et al., 2022). Cuprotosis can affect the polarization and function of tumor-associated macrophages (TAMs), which are immune cells that play a crucial role in the tumor microenvironment. TAMs can exhibit either pro-tumor (M2-like) or anti-tumor (M1-like) phenotypes. Cuprotosis has been shown to promote the polarization of TAMs toward the M1-like phenotype, which is associated with anti-tumor activity. This shift in TAM polarization can lead to a more favorable tumor microenvironment for immune responses. All these results suggested that cuprotosis may have a role in reshaoing tumor microenvironment and regulate efficacy of immunotherapy.

## The strategies of copper-associated treatment

### Copper-based compounds

Copper compounds have been studied for their potential use in cancer treatment, as cancer cells are known to have a higher copper uptake and retention compared to normal cells. The idea behind using copper compounds in cancer treatment is to selectively target cancer cells by inducing excessive copper accumulation in them, leading to cuprotosis. Several copper compounds have been studied for their ability to induce cell death in cancer cells.

#### Copper oxide nanoparticles

CuO NPs are nanoparticles composed of copper and oxygen atoms (Singh et al., 2016). They have a high surface area-to-volume ratio, which allows them to interact more efficiently with cells and tissues. When CuO NPs enter cancer cell, they can interact with intracellular copper ions and generate ROS, such as hydrogen peroxide (H2O2) and superoxide anion (Meghana et al., 2015). These ROS can then react with cellular molecules, such as lipids, proteins, and DNA, and cause oxidative damage and disrupt mitochondrial function. CuO NPs have been found to induce cell death in breast cancer, colorectal cancer, prostate cancer, lung cancers and glioblastmoma multiforme (GBM) in preclinical studies (Wang et al., 2017; Elsayed et al., 2021; Tabrez et al., 2022; Zhao et al., 2022; Zughaibi et al., 2022).

#### Copper-bis(thiosemicarbazone) complexes

These complexes consist of a copper ion coordinated with two thiosemicarbazone ligands, which can bind to intracellular copper and induce oxidative stress and disruption of copper homeostasis, ultimately leading to cancer cell death. A copper complex called Casiopeína III-ia has been shown to selectively induce cuprotosis in breast cancer cells and lung cancer cells and inhibit tumor growth in mice (Davila-Manzanilla et al., 2017). Another study showed that Casiopeína III-ia inhibibited migration and invasion of prostate cancer cells (Krasnovskaya et al., 2020).

#### Copper (II) complexes of curcumin

They are copper-containing compounds that consist of a copper ion coordinated with curcumin ligands. Copper (II) complexes of curcumin have been shown to have enhanced anti-cancer activity compared to curcumin alone due to their ability to induce cancer cell death via multiple mechanisms, including the generation of reactive oxygen species, inhibition of cell proliferation, and induction of apoptosis. Studies have shown that copper (II) complexes of curcumin can selectively target cancer cells while sparing normal cells, indicating their potential as cancer therapeutics with reduced toxicity. Additionally, these complexes have shown promising activity against a variety of cancer types, including breast cancer, lung cancer and colon cancer (Lou et al., 2010; Luo et al., 2017; Agarwal et al., 2018).

#### Copper-doxorubicin conjugates

The goal of these conjugates is to improve the efficacy and reduce the toxicity of doxorubicin by enhancing its selectivity and delivery to cancer cells via multiple mechanisms, including DNA damage, inhibition of topoisomerase II, and generation of reactive oxygen species. Study showed that the hybrid nanoparticles had increased cytotoxicity against liver cancer cells compared to doxorubicin alone, the hybrid nanoparticles were found to selectively accumulate in cancer cells due to the targeting effect of folic acid while sparing normal cells (Xu et al., 2020).

However, the clinical translation of copper-based cancer treatment using copper compounds faces several challenges, including the potential toxicity of copper accumulation in healthy tissues and the heterogeneity of copper accumulation among different types of cancer cells. Maybe further the study can focus on the drug delivery system to enhance the safety and efficacy of copper-based cancer treatment.

### Targeting copper-dependent enzymes

#### Lysyl oxidase (LOX)

It is a Copper-dependent enzyme that is involved in the cross-linking of collagen and elastin fibers in the extracellular matrix, which is important for the formation and stabilization of blood vessels. LOX has been shown to promote tumor growth and metastasis by promoting angiogenesis and enhancing the invasiveness of cancer cells. Therefore, inhibition of LOX activity using copper chelators or other inhibitors has been investigated as a potential anti-cancer strategy. Studies have shown that inhibition of LOX activity using copper chelators or other inhibitors can reduce tumor growth and metastasis in various cancer types. Copper chelator tetrathiomolybdate inhibited LOX activity and demonstrated antiangiogenic, antifibrogenic and anti0inflammatory actions in a mouse model of breast cancer (Cox et al., 2016). Similarly, an LOX inhibitor called β-aminopropionitrile reduced lung metastasis in cervical cancer (Yang et al., 2013). In addition, LOX has been shown to be involved in the formation of a pre-metastatic niche, a microenvironment that promotes the growth of metastatic cancer cells in distant organs. LOX inhibition using a copper chelator reduced the formation of a pre-metastatic niche and prevented the growth of metastatic breast cancer cells in the lung (Sceneay et al., 2013).

#### Superoxide dismutase (SOD)

It is a copper-dependent enzyme that plays an important role in protecting cells from oxidative stress (Bresciani et al., 2015). In cancer treatment, one strategy for targeting SOD in cancer treatment involves the use of SOD inhibitors, which can increase the levels of reactive oxygen species (ROS) in cancer cells and induce cell death. However, the development of effective SOD inhibitors has been challenging due to the complex structure and function of the enzyme. Another approach to targeting SOD in cancer treatment involves the use of SOD mimetics such as Mn porphyrins (Batinic-Haberle and Tome, 2019). Another mimic, MnTE-2-PyP, the researchers found that treatment with MnTE-2-PyP inhibited the growth of lung cancer cells both *in vitro* and, combining with the chemotherapy drug cisplatin resulted in greater inhibition of tumor growth compared to either treatment alone (Yakovlev et al., 2010).

#### Dopamine hydroxylase (DBH)

It is a copper-dependent enzyme that converts dopamine to norepinephrine, a neurotransmitter and hormone that plays a role in the stress response. While DBH is primarily involved in the regulation of the nervous system, it has also been implicated in cancer (Chakroborty et al., 2004). One possible mechanism for DBH’s role in cancer is its ability to regulate the levels of reactive oxygen species (ROS) in cells. DBH has been shown to modulate ROS levels through its interaction with copper ions (Tsang et al., 2021).

### Copper’s proteasome-inhibitory anticancer strategy

It refers to the use of copper-chelating agents such as clioquinol and tetrathiomolybdate to disrupt the function of proteasomes in cancer cells (Zhang et al., 2017). Proteasomes are large protein complexes that play a critical role in the degradation and recycling of intracellular proteins, including those involved in cell cycle regulation, DNA repair, and apoptosis. Cancer cells are known to have high levels of proteasome activity, which helps them to survive and proliferate. By chelating copper ions, clioquinol and tetrathiomolybdate can inhibit proteasome activity in cancer cells, leading to the accumulation of toxic protein aggregates and ultimately causing cell death (Wehbe et al., 2017). Additionally, the accumulation of toxic protein aggregates can also enhance the sensitivity of cancer cells to chemotherapy and radiation therapy. There are several clinical trials involving copper-chelating agents in cancer patients such as NCT01837329, NCT00150995, NCT00176800, NCT00352742 and NCT00383851. These trials are investigating the safety and efficacy of copper-chelating agents in combination with chemotherapy, immunotherapy, or other agents for the treatment of various types of solid tumors. The results of these trials will help to determine the potential of copper-chelating agents targeting cooper-dependent enzymes as a novel approach to cancer therapy.

## Discussion

In the past several decades, the development of new cancer therapies has been an ongoing endeavor in the targeting therapy field. Metal ions such has iron, copper and zinc were confirmed to play important roles in various cellular process of cancer, including DNA synthesis, energy production and cell signaling. Imbalances in metal ion hemeostasis can induce irreversible cell damage and cell death. Ferroptosis has been gaining significant attention in recent years as a potential cancer strategy, due to its unique mechanism of targeting cancer cells that are resistant to classic cell death such as apoptosis. Cancer cells often have higher levels of iron and are more susceptible to ferroptosis than normal cells (Tang et al., 2021). This selective targeting of malignant cells while sparing normal cells may help to minimize side effects and improve the overall effectiveness of cancer treatment. Inspired by the discovery of ferroptosis, further studies have identified another metal ion metabolism abnormality, known as “cuprotosis,” which has gained significant attention as a potential cancer treatment strategy. Just like iron, copper levels are markedly higher in tumor cells compared to normal cells (Babak and Ahn, 2021). Hence, targeting cuprotosis in cancer therapy provides similar benefits as ferroptosis.

One approach to targeting copper is through copper chelation therapy. By binding to copper ions, chelating agents can reduce the level of copper in cancer cells and inhibit their growth. Tetrathiomolybdate and trientine (used to treat Wilson’s disease) are two examples of copper chelating agents that have been studied in preclinical and clinical trials for their anti-cancer activity. Tetrathiomolybdate has been shown to have anti-angiogenic effects and inhibit the growth of several types of cancer, including breast (phase II clinical trials), prostate cancer (phase II clinical trials) and ovarian cancer (Henry et al., 2006; Kim et al., 2012; Chan et al., 2017).

Another approach to targeting copper is through inhibiting cuprotosis-related genes or proteins. FDX1 is the key gene that contributes to cuprotosis. Study showed knockdown of FDX1 resulted in the downregulation of cuprotosis in kidney renal clear tumor cells (Xu et al., 2022). FDX1 is found to be highly expressed in gliomas and associated with worse prognosis. Interestingly, FDX1 expression is positively correlated with the infiltration of immune cells. This suggests that cuprotosis may not only have potential as a therapeutic target for cancer but may also have implications in the modulation of the immune system (Zhang et al., 2023). By inhibiting this key factor, the production of ROS can be reduced, preventing cell death. Inhibitors of FDX1 or LIAS have been studied for their anti-cancer activity, and have shown promising results in preclinical studies (Xie et al., 2022).

Combination therapy with cuprotosis inhibitors and other anti-cancer agents has also shown promising results in preclinical studies. Combination therapy with cuprotosis inhibitors and other anti-cancer agents has shown promising results in preclinical studies. For example, combining the copper chelating agent tetrathiomolybdate with cisplatin in a mouse model of ovarian cancer led to a significant reduction in tumor growth compared to either agent alone (Kim et al., 2012). Similarly, combining the cuprotosis inhibitor elesclomol with paclitaxel in a preclinical model of breast cancer enhanced the efficacy of both drugs, resulting in increased cancer cell death (Zheng P. et al., 2022).

Moreover, immune modulation with cuprotosis inhibitors and immunotherapies can have a synergistic effect in cancer treatment. Combination therapy with cuprotosis inhibitors and immunotherapies can have a synergistic effect in cancer treatment. Cuprotosis can release DAMPs, which can activate the immune system and enhance the efficacy of immunotherapies. Checkpoint inhibitors can block inhibitory signals and enhance T cell activation, while chimeric antigen receptor T cell (CAR-T) therapy can directly target cancer cells. Cancer vaccines can also enhance the immune response against cancer cells. Therefore, combining these immunotherapies with cuprotosis inhibitors can lead to a more effective and comprehensive cancer treatment approach.

Although the development of copper-targeting agents for cancer therapy is still in its early stages, the promising results of preclinical studies suggest that this approach has the potential to revolutionize cancer treatment. Further research is needed to optimize the therapeutic efficacy and safety of these agents, but targeting copper metabolism is an exciting avenue for the development of new cancer therapies.

## Abbreviations

ACD, accidental cell death; PCD, programmed cell death; NARCD, non-apoptotic regulated cell death; ROS, reactive oxygen species; TCA cycle, tricarboxylic acid cycle; CTR1, copper transporter 1; SLC7A11, solute carrier family 7 member 11; VEGF, vascular endothelial growth factor; TAMs, tumor-associated macrophages; DAPMs, damage-associated molecular patterns; H2O2, hydrogen peroxide; GBM, glioblastmoma multiforme; LOX, Lysyl oxidase; SOD, Superoxide dismutase; DBH, Dopamine hydroxylase; CAR-T, chimeric antigen receptor T cell.
